# A Diagnostic Conundrum: Tuberculosis or Salmonella Neck Abscess?

**DOI:** 10.7759/cureus.37224

**Published:** 2023-04-06

**Authors:** Isaac Yieng Ler Tan, Hardip Gendeh, Marina Mat Baki, Reena Rahayu Md Zin, Bee See Goh

**Affiliations:** 1 Department of Otorhinolaryngology - Head and Neck Surgery, Faculty of Medicine, Universiti Kebangsaan Malaysia Medical Centre, Kuala Lumpur, MYS; 2 Department of Pathology, Faculty of Medicine, Universiti Kebangsaan Malaysia Medical Centre, Kuala Lumpur, MYS

**Keywords:** infectious disease pathology, tuberculosis, salmonella nontyphi, neck abscess, granuloma

## Abstract

Granulomatous neck abscesses are commonly associated with tuberculosis (TB). These chronic inflammatory reactions are rarely seen in *Salmonella non-typhi* (SN) infections. We report two cases of SN granuloma presenting as neck abscesses in poultry farmers. TB* *polymerase chain reactions (PCR) were negative. Histopathology reported necrotizing granulomatous inflammation. *Salmonella* species are known to cause true granulomas in bone marrow, liver, and spleen. To the best of our knowledge, true granulomas have not been described in cervical lymph nodes. The aim of this report was to highlight the importance of considering other causative microbiological agents in cases of granulomatous neck abscesses. The patients recovered after receiving treatment with surgical drainage and intravenous antibiotics.

## Introduction

*Salmonella non-typhi *(SN) species are foodborne bacteria that cause gastroenteritis, bacteremia, and focal infections. Extraintestinal manifestations of the infection are reported in 8-16.7% of 93 million total cases annually worldwide [1]. SN can spread to every anatomical site via hematogenous seeding after bacteremia. However, neck abscesses can develop via the local spread, but these infections are infrequently described [2,3].

Granulomatous reactions are rarely seen in SN infections outside the bone marrow, liver, and spleen [3]. To the best of our knowledge, true granulomas have not been described in cervical lymph nodes. In countries with a high incidence of TB, it must be excluded among other differential diagnoses of granulomatous abscesses such as leprosy and cat-scratch disease [4]. We present a case series involving two diabetic patients with cervical abscesses caused by SN infection in Malaysia. We aim to highlight the importance of considering other causative microbiological agents in cases of granulomatous neck abscesses. The management of SN neck abscesses is also discussed.

## Case presentation

Case 1

A 62-year-old healthy female sought treatment at a general practitioner for right-sided neck swelling that had been gradually increasing in size and turned painful over a fortnight. The swelling had been preceded by fever associated with chills, rigor, myalgia, and arthralgia. She was given two weeks of amoxicillin (500 mg every eight hours) but her symptoms failed to resolve. She had no symptoms of upper airway obstruction and denied loss of weight or appetite or any prolonged cough or previous contact with TB patients. She is a poultry farm worker. Her past and familial medical histories were unremarkable.

On examination, the swelling was located over the right angle of the mandible, measuring 5 x 4 cm with no overlying skin changes or punctum, and bordered by the ear lobule superiorly, right sternocleidomastoid posteriorly, and by masseter anteriorly. Intraoral examination showed poor dentition and flexible nasopharyngolaryngoscopy showed no pharyngeal medialization.

Laboratory analysis revealed an elevated leukocyte count of 14,000/uL, predominantly consisting of 86% neutrophils, with an increased C-reactive protein (CRP) concentration at 50.1 mg/L. Additionally, the patient was identified as having poorly managed diabetes mellitus, as indicated by raised glycosylated hemoglobin levels. Her human immunodeficiency virus (HIV) screening was also non-reactive. A contrasted CT of the neck revealed a rim-enhancing lesion within the right sternocleidomastoid muscle measuring 3.3 x 3.1 x 4 cm. The muscle was bulky and streaky. The adjacent right carotid and internal jugular vessels were displaced medially. 

**Figure 1:**
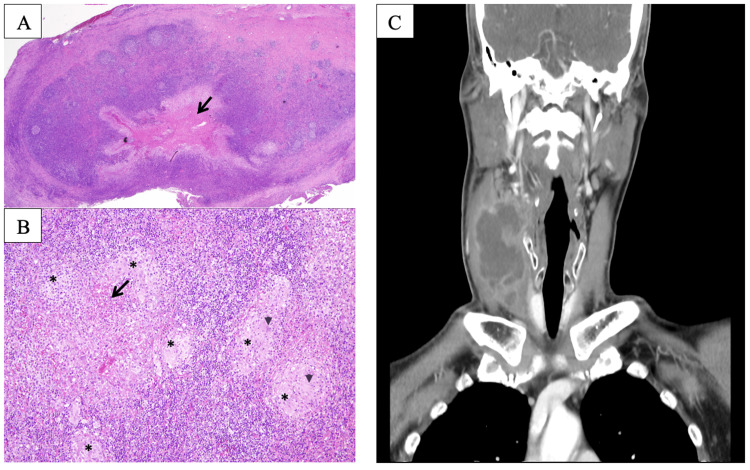
Photomicrographs of Case 1 and CT imaging of Case 2 (A) H&E stain, 20x shows a section from the cervical lymph node showing effacement of normal architecture by chronic granulomatous inflammation with central necrosis (arrow). (B) H&E, 100x. High magnification showing granulomas (asterisks) consisting of epithelioid histiocytes surrounded by chronic inflammatory cells with central necrosis (arrow). Occasional multinucleated giant cells are seen (arrowheads). Ziehl-Neelsen stain does not highlight any acid-fast bacilli and the PAS stain is negative for fungal bodies. (C) CT imaging of the neck of Case 2 demonstrated a multiloculated collection in the right posterior cervical space with a hypodense center CT: computed tomography

The patient was hospitalized, and antibiotics were administered alongside therapy for diabetes mellitus including insulin injections. Amoxicillin-clavulanic acid (1.2 g every eight hours) was administered and she underwent an urgent incision and drainage. Postoperatively, her condition improved with antimicrobial administration and daily irrigation of the wounded area. Histopathology studies reported epithelioid histiocytes surrounded by chronic inflammatory cells with central necrosis. Occasional multinucleated giant cells were seen. Ziehl-Neelsen stain and PAS stain were negative (Figures 1A, 1B).

The infectious disease team was consulted, and a TB polymerase chain reaction (PCR) was performed. Her antibiotics were stopped, and she was treated preemptively for tuberculosis lymphadenitis while awaiting confirmation of TB. However, the neck wound did not improve during the course of antituberculosis treatment. Subsequently, TB PCR returned negative while SN was isolated, which was sensitive to ciprofloxacin, co-trimoxazole, ceftriaxone, and ampicillin. She was started on intravenous ciprofloxacin (200 mg every 12 hours) for three more days. The patient was discharged after one week following surgery with symptom abatement. She completed a 14-day course of oral ciprofloxacin while maintaining good sugar regulation.

Case 2

A previously healthy 42-year-old male presented with a two-week history of right-sided cervical swelling and associated discomfort. The patient reported febrile episodes accompanied by chills, rigors, and night sweats, without any signs of upper airway obstruction. He denied any recent neck trauma, weight loss, dental pain, or dental procedures. Although he had not experienced any abdominal pain or diarrhea in the preceding month, he did report increased thirst and frequent urination. No history of unpasteurized milk intake or exposure to undercooked meat was noted. He works as a poultry farm driver. There had been no significant travel, TB exposure, or intravenous drug use. He was tachycardic and febrile (38.5 °C), and physical examination revealed a tender, erythematous, non-fluctuant 6 × 7 cm mass in the right neck extending from the angle of the mandible. No other lymphadenopathy was identified. Oral cavity examination revealed fair dentition.

Initial laboratory studies were remarkable for a white blood cell count of 17.0 × 10/dL, with 79% neutrophils, and serum glucose of 18 mmol/L. Chest X-ray revealed clear lung fields. CT imaging of the neck demonstrated a 4 × 7 cm multiloculated mass in the right posterior cervical space with a hypodense center. The collection extended from the right submandibular gland to the clavicular head of the sternocleidomastoid below in the right anterior cervical space with a mass effect to the right internal jugular vein (Figure 1C). Intravenous amoxicillin-clavulanic acid (1.2 g every eight hours) was administered and he underwent urgent incision and drainage. Thirty milliliters of purulent discharge were evacuated. 

Histopathology studies reported epithelioid histiocytes surrounded by chronic inflammatory cells with central necrosis. Occasional multinucleated giant cells were seen. Ziehl-Neelsen and PAS stain were negative. Stool cultures and HIV tests were also negative. The patient's hemoglobin A1c was 13.6%. TB PCR was negative. SN was isolated, which was sensitive to ciprofloxacin, co-trimoxazole, ceftriaxone, and ampicillin. 

The patient was started on intravenous ciprofloxacin (200 mg every 12 hours) for a further three days. He was discharged home after five days with complete recovery after achieving good glycemic control, and two weeks of oral ciprofloxacin was prescribed.

## Discussion

Head and neck infections are usually polymicrobial, with only 5% of cases being purely aerobic and 25% with isolated anaerobes [5]. Negative intraoperative cultures are usually attributed to early antimicrobial treatment administered prior to surgical intervention. 

Aerobic bacteria that are typically isolated include *Streptococcus viridans, *coagulase-negative staphylococci (CoNS), and *Staphylococcus aureus*. *Klebsiella pneumoniae* has been identified as the most frequently encountered aerobic organism. In terms of anaerobes, *Bacteroides*, *Peptostreptococcus, Fusobacterium*, and *Prevotella* are the most predominant, in decreasing order of prevalence [6]. Moreover, there have been infrequent accounts of neck abscesses resulting from SN infection in individuals with pre-existing systemic conditions, such as diabetes mellitus, liver disease, or malignancies [1].

The manifestation of a *Salmonella* abscess is determined by the mode of transmission, which is either from hematogenous or lymphatic pathways. Upon oral inoculation, *Salmonella* species has the potential to invade tonsillar tissues, subsequently causing lymphadenitis along the cervical lymphatic network [1]. Both our patients had exposure to poultry. Undercooked poultry is the most important reservoir of *Salmonella*. Contaminated food left in ambient temperatures is a potential vehicle for infection. In Malaysia, the prevalence of *Salmonella* in raw and cooked foods was reported to be 23% in 1995. However, vigorous public health measures have been implemented, leading to the reduction of the average annual incidence to 1.74 per 100,000 population [7]. Our patients had poor dental hygiene but showed no symptoms of gastrointestinal infection and had negative blood and stool cultures. This suggests that the organism seeded orally via lymphatic tissues such as the tonsils or traveled along the lymphatic system to the submandibular space [6].

Empiric antimicrobial treatment with a combination of penicillin plus a beta-lactamase inhibitor, e.g., amoxicillin/clavulanate, is recommended among other options to provide enough coverage for both anaerobic and aerobic bacteria [8]. Certain cases require surgical exploration and drainage of infection. Intraoperative cultures must be taken to establish the microbiological etiology and to evaluate antibiotic sensitivity to tailor the antibiotic treatment. The majority of invasive SN cases were empirically treated by at least one appropriate antimicrobial, often a third‐generation cephalosporin (ceftriaxone or cefotaxime) or a fluoroquinolone (ciprofloxacin or levofloxacin) within 72 hours of presentation. Current guidelines dictate that the duration of neck abscess is shorter compared to other sites of infection, a minimum of two weeks and four to six weeks, respectively [9].

Our patients' histopathology reports revealed epithelioid histiocytes surrounded by chronic inflammatory cells and central necrosis with occasional multinucleated giant cells, in keeping with chronic granulomatous inflammation. Special stains with Ziehl-Neelsen stain and PAS were negative for tubercle bacilli and fungal elements respectively. Mantoux test and TB PCR also ruled out tuberculous infection. The usual histological findings in *Salmonella* lesions are necrosis and histiocytic proliferation with prominent lymphophagocytosis. Granulomatous inflammation is an unusual histopathological presentation and when present, the granulomas of *Salmonella* are typically found in the bone marrow and gastrointestinal tract [3,9].

Granuloma formation represents a pervasive chronic inflammatory reaction in response to enduring endogenous or exogenous stimuli.Common microorganisms recognized as etiological agents of granulomatous lymphadenitis include *Mycobacterium tuberculosis, *atypical *Mycobacteria Histoplasma *species*, Toxoplasma *species, and cat scratch* Bacillus*. The majority of granulomatous lymphadenitis can be attributed to atypical* Mycobacteria *[6]. Enteric bacilli such as SN, which infrequently cause lymphadenitis in a healthy host, may function as chronic irritants and trigger granuloma formation in individuals with phagocytic compromise [10].

We postulate that our patients' prior extended fever was evidence of the presence of *Salmonella* bacteremia. Given that *Salmonella* species can inoculate the body through the oral route, it serves as a gateway to the cervical soft tissues via parapharyngeal lymphatic tissue. The development of granulomas could potentially stem from chronic bacterial irritation within the lymph node, subsequently leading to suppuration and abscess formation [10].

## Conclusions

The present case series highlights the unusual presentation of Salmonellosis with a chronic granulomatous response on histology. TB must be excluded at the outset in the differential diagnosis of granulomatous abscesses in regions of high TB prevalence. However, SN infection should be considered in immunocompromised patients presenting with neck abscesses of unclear etiology.
